# Application of artificial intelligence-based dual source CT scanning in the differentiation of lung adenocarcinoma in situ and minimally invasive adenocarcinoma

**DOI:** 10.12669/pjms.40.3.8454

**Published:** 2024

**Authors:** Lihong Liu, Zhihua Ni, Jian Zhang, Junsong Zhao, Jieyun Shen

**Affiliations:** 1 Lihong Liu, PhD. Department of Radiology, Shanghai Baoshan Hospital of Integrated Traditional Chinese and Western Medicine, Shanghai, China; 2 Zhihua Ni, PhD. Department of Radiology, Shanghai Baoshan Hospital of Integrated Traditional Chinese and Western Medicine, Shanghai, China; 3 Jian Zhang, PhD. Department of Radiology, Shanghai Universal Medical Imaging Diagnostic Center, Shanghai, China; 4 Junsong Zhao, PhD, Department of Radiology, Shanghai Baoshan Hospital of Integrated Traditional Chinese and Western Medicine, Shanghai, China; 5 Jieyun Shen, PhD, Department of Radiology, Shanghai Universal Medical Imaging Diagnostic Center, Shanghai, China

**Keywords:** Artificial intelligence, Dual source CT scanning, Lung adenocarcinoma in situ, Minimally invasive adenocarcinoma

## Abstract

**Background and Objective:**

Lung adenocarcinoma is the most common type of lung cancer with highly incidence and mortality. Due to the overlap of morphological features, it is difficult to distinguish clinically between preinvasive lesions (in situ adenocarcinoma, AIS) and invasive lesions (minimally invasive adenocarcinoma, MIA), which appear as ground glass cloudy nodules. This study was performed to probe the application value of artificial intelligence (AI)-based dual source CT scanning in the differentiation of AIS as well as MIA.

**Methods:**

The clinical data of 136 patients in Shanghai Baoshan Hospital of Integrated Traditional Chinese and Western Medicine from January 2019 to January 2022 were retrospectively analyzed. The accuracy of AI in distinguishing lung AIS (n=76) and MIA (n=60) were analyzed. The effectiveness of AI in detecting nodules and its diagnostic efficacy for AIS and MIA were explored.

**Results:**

The proportion of patients with clear and regular lesion boundaries in AIS was higher than that in MIA. The mean lesion diameter of AIS patients was shorter than MIA patients. There was no difference in the CT value between AIS and MIA in the ground glass nodule density area of pure ground glass nodule and mixed ground glass nodule, but the CT value of the solid nodule density area in AIS was lower. The occurrence of pulmonary vascular abnormality, air bronchogram sign, and pleural depression in AIS patients were lower than MIA patients. The detection rate of AI for lung adenocarcinoma with nodule diameter ≤ 5 mm, complete solid nodules and ground glass nodules was significantly higher than radiologists. The sensitivity, specificity, positive prediction rate, negative prediction rate and accuracy of AI detection were significantly higher than radiologists.

**Conclusion:**

AI-based dual source CT scanning can clearly show the morphological characteristics of lung adenocarcinoma, which is helpful for the differential diagnosis of lung AIS as well as MIA.

## INTRODUCTION

Lung cancer is one of the most common malignant cancer diseases worldwide, ranking first in both incidence and mortality.^1^ Based on histology, lung cancer is divided into two main subtypes: small cell lung carcinoma (SCLC) and non-small-cell lung carcinoma (NSCLC), accounting for 15 and 85% of all cases, respectively.^2^ NSCLC is further classified into three types: squamous-cell carcinoma, adenocarcinoma, and large-cell carcinoma.^3^ Lung adenocarcinoma is the most common type of lung cancer, and comprises around 40% of all lung cancer cases.^4^ Patients with lung adenocarcinoma have a typical trend of diversity, both in histopathological classification and molecular biomarkers.^5^ Therefore, if the condition of the patient can be confirmed and effective intervention measures can be taken at this stage, it is crucial to ensure the prognosis of the patient.

Lung adenocarcinoma originates from the bronchial mucosa epithelium, and is divided into preinvasive lesions, minimally invasive adenocarcinoma (MIA) and invasive adenocarcinoma.^6^ Preinvasive lesions are classified into atypical adenomatous hyperplasia and adenocarcinoma in situ (AIS).^7^ Lung CT is a crucial basis for early lung cancer screening.^8^ The CT examination of lung adenocarcinoma often presents ground glass nodules, with slightly increased density in the pulmonary window and visible bronchi and blood vessels in the area with clear edges. The occurrence of this image manifestation is a non-specific indication, and AIS and MIA both can exhibit this imaging feature.^9^ In conventional CT plain scan and enhanced scan, patients not only receive more radiation doses, but also often have the problem of the same appearance of different diseases in imaging diagnosis. Morphological similarity does not mean that they are essentially the same, and only nodular lesions with typical morphological and density characteristics can be definitively diagnosed.^10^ Therefore, the nature of lung nodules cannot be determined by morphological analysis only. Dual source CT uses two X-ray generators and two detectors to capture CT images. The cutting-edge energy spectrum purification technology and dual-energy imaging technology of the equipment significantly increase the tissue identification ability of the machine, eliminate the image registration problem, and make the imaging clearer and more accurate to distinguish different lesions of different human tissues.^11^ The diagnostic application of dual source CT has been widely reported in various diseases, such as head and neck cancer and congenital heart disease.^12^,^13^

Recently, the development of artificial intelligence (AI) has created many convenience for doctors to perform automatic detection, measurement, along with risk assessment of lung cancers.^14^ The application of AI data calculation to CT reading is an effective, fast, and accurate shortcut to screen different types of lung cancer.^15^ AI-based dual source CT scanning can provide images without motion artifacts and unprecedented clarity. The rapid scanning of the system allows for more accurate measurements of lesions, improves atherosclerotic plaque and stent imaging, and enhances functional assessment.^16^ AI can quickly detect lung nodules and predict benign and malignant in many CT images, but the accuracy of its prediction is unclear.

In addition, it is of great clinical significance to accurately distinguish AIS and MIA before operation. AIS can be followed up without hasty surgical resection, in order to exclude atypical adenomatous hyperplasia or other benign lesions, not only to avoid overtreatment, and does not affect the survival rate of patients. MIA needs to be surgically removed to prevent the development of invasive cancer and distant metastasis.^17^ Therefore, in this research, the application of AI-based dual source CT scanning in the differentiation of AIS and MIA was explored.

## METHODS

The clinical data of 136 patients with lung adenocarcinoma diagnosed and confirmed by follow-up in our hospital from January 2019 to January 2022 were retrospectively analyzed.

### Inclusion criteria:

- Age >18 years old.
- The disease was confirmed by histopathological examination.
- All patients underwent AI-based dual source CT examination and the examination imaging data were complete.

### Exclusion Criteria:

- Patients with pulmonary tuberculosis.
- Other tumors.
- Severe heart, liver as well as kidney dysfunction, other lung diseases.
- With lung adenocarcinoma and lymph node. (5) Pleural and other distant metastasis.
- There were 67 males and 69 females. The mean age was 53.58±5.21 years, ranging from 35-68 years.

### Ethical Approval

This study was approved by the medical ethics Committee of Shanghai Universal Medical Imaging Diagnostic Center (approval number: SHQJ-2022-12) on 27^th^ December 2022, and all patients signed informed consent.

The examination instrument was Siemens SOMATOM Definition Flash dual-source CT. All the patients underwent pathological diagnosis, conventional CT plain scan and dual-source CT dual-energy diagnosis. Siemens SOMATOM Definition Flash dual-source CT was used. The bulb voltage was set to 100 kvp and 140 kvp respectively, and the bulb current was set to 230 mAs and 172 m respectively. The FOV was set at 50 cm × 50 cm and 33 cm × 33 cm, and the scanning Angle was set at 94°C. The CARE Dose 4D dynamic exposure dose regulator was started. The collimation, rotation speed and sweep pitch were set to 0.6 mm × 128 mm, 0.5 s/week and 0.6, respectively. Routine plain scan was performed first. The scanning staff instructed the patient to inhale and exhale correctly, set the scanning layer thickness to 5 mm, and scan the thoracic entrance to the diaphragmatic area. The scanning layer thickness and scanning range of dual-energy enhanced scan were the same as that of conventional scan. Iohexol was used as the contrast agent. According to the dose of 350 mgI/mL, the scanning flow rate was set to 3.0 mL/s, and the scanning delay was 35s.

### Image processing and diagnosis

The AI auxiliary screening system for pulmonary nodules was provided by InferRead CT Lung. Two groups of images were transferred into the post-processing workstation and processed by AI system. For the detection and qualitative diagnosis of pulmonary nodules, an attending physician first detected nodules in high-resolution thin-slice chest CT images and diagnosed them as benign possible or suspicious malignant nodules according to the size, density, morphology and relationship with peripheral blood vessels and bronchi, and then reviewed by the associate chief physician. The other two radiologists with senior professional titles combined artificial intelligence and reviewed imaging reports to detect and identify nodules. When they disagreed, they combined multi-planar reconstruction and discussed to obtain consistent results. And it was used as the gold standard for true nodule detection.

### Observation indexes

The Siemens SOMATOM Definition Flash dual-source CT images of lung AIS and MIA were observed and compared, including the occurrence site, shape, edge, density, lesion diameter and CT value, pulmonary vascular abnormality, air bronchogram sign, as well as pleural depression sign in the lesion.

### Statistical analysis

SPSS 22.0 was adopted to process the data. Counting data were exhibited as [n (%)], and χ^2^ test was implemented for comparison between groups. Measurement data were expressed as the mean ± standard deviation (SD) and the comparison of groups was tested by the independent-t test. According to the results, the receiver operating characteristic curve (ROC) was drawn to obtain the area under the curve, sensitivity, and specificity at different cut-off points.^18^ P<0.05 was statistical significance.

## RESULTS

All patients were confirmed by histopathology, including 76 cases of lung AIS and 60 cases of MIA. There are 48 (63.16%) cases of highly differentiated, 18 (23.68%) cases of moderately differentiated, and 10 (13.16%) cases of poorly differentiated in AIS group. There are 40 (66.67%) cases of highly differentiated, 15 (25.00%) cases of moderately differentiated, and five (8.33%) cases of poorly differentiated in AIS group. The pathological results revealed no significant difference in the pathological differentiation degree between lung AIS and MIA patients (P>0.05).

It is shown in Table-I that, nodules mostly occurred in the upper lobes of the two lungs in the AIS group and MIA group, accounting for 73.68% (56/76) and 73.34% (44/60), respectively, and no significant difference was observed between the two groups (P>0.05). The proportions of patients with clear and regular lesion boundaries in AIS were 73.68% (56/76) and 76.32% (58/76) respectively, which were significantly higher than those in patients with MIA 21.67% (13/60) and 25.00% (15/60), and the difference was statistically significant (P<0.05).

**Table-I:** Occurrence site, shape, and edge analysis of patients with lung AIS and MIA (case, %).

Morphological characteristics of lesions	AIS (n=76)	MIA (n=60)	P
***Occurrence site***			>0.05
Upper right	36 (47.37)	30 (50.00)	
Low right	10 (13.16)	8 (13.33)	
Middle right	6 (7.89)	5 (8.33)	
Upper left	20 (26.32)	14 (23.34)	
Low left	4 (5.26)	3 (5.00)	
***Shape***			
Regular	58 (76.32)	15 (25.00)	<0.05
Irregular	18 (23.68)	45 (75.00)
***Edge***			
Distinct	56 (73.68)	13 (21.67)	<0.05
Vague	20 (26.32)	47 (78.33)	

**Fig.1:**
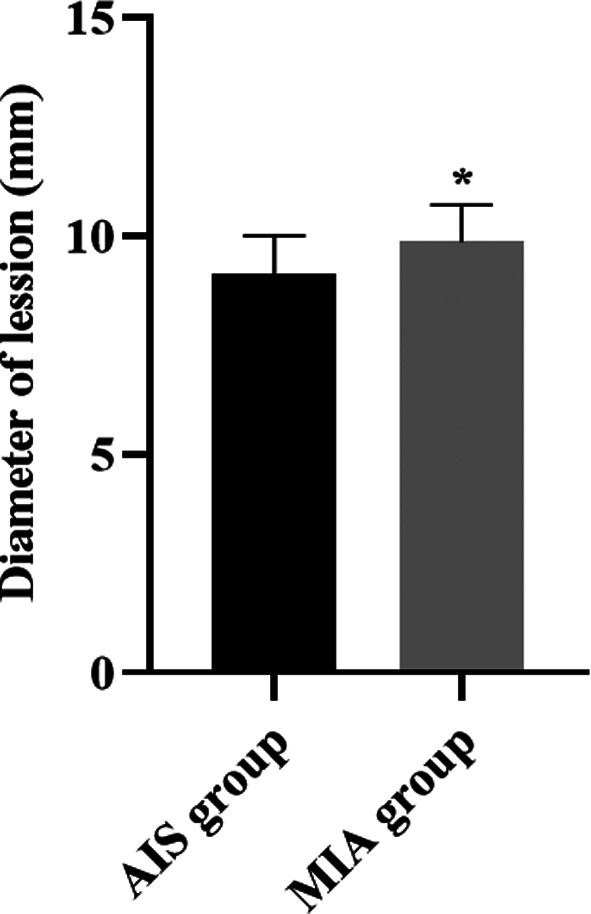
Diameter of lesion in lung AIS and MIA patients. *P<0.05.

There are 52 (68.42%) cases of pure ground glass nodule, 24 (31.58%) cases of mixed ground glass nodules, and 0 (0.00%) case of solid nodules in AIS group. There were 16 (26.67%) cases of pure ground glass nodule, 42 (70.00%) cases of mixed ground glass nodules, and 2 (3.33%) case of solid nodules in MIA group. There was a significant difference in the density types of the two lesions between the two groups (χ^2^=23.38, P<0.05). It was indicated in Fig.1 that; the mean lesion diameter of AIS patients was shorter compared to MIA patients (P<0.05).

**Fig. 2:**
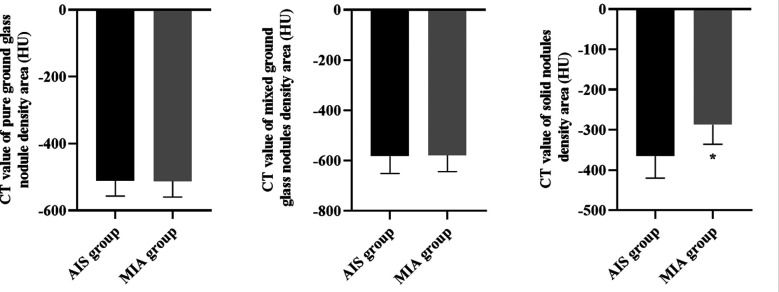
CT values of different components of AIS and MIA. *P<0.05.

It is indicated in Fig.2 that, there was no difference in the CT value between AIS and MIA in the ground glass nodule density area of pure ground glass nodule and mixed ground glass nodule (P>0.05), but the CT value of the solid nodule density area in AIS was significantly lower than that in MIA (P<0.05).

The incidence of pulmonary vascular abnormality, air bronchogram sign as well as pleural depression in lung AIS patients were lower compared to MIA patients. P<0.05 (Table-II,)

**Table-II:** Occurrence of pulmonary vascular abnormality, air bronchus sign, as well as pleural depression sign in the lesion (case, %).

Groups	Cases	Pulmonary vascular abnormality	Air bronchogram sign	Pleural depression sign
AIS	76	7 (9.21)	4 (5.26)	5 (6.58)
MIA	60	13 (21.67)	11 (18.33)	16 (26.67)
*χ* ^2^		4.15	5.84	10.36
*P*		<0.05	<0.05	<0.05

A total of 170 true nodules were detected by two senior radiologists combined with artificial intelligence in CT of 136 patients (Table-III). The comparison of malignant detection rates between AI and radiologist showed that the detection rate of AI for lung adenocarcinoma with nodule diameter ≤5 mm, complete solid nodules and ground glass nodules was significantly higher than that of radiologists (P<0.05).

**Table-III:** Comparison of number of nodules detected by AI and radiologist (case, %).

Nodule classification		n	AI	Radiologists	χ^2^	P
Nodule diameter	≤5 mm	48	46 (95.83)	23 (47.92)	27.26	<0.05
	5-10	68	66 (97.06)	61 (89.71)	2.98	0.08
	≥10 mm	54	53 (98.15)	51 (94.44)	1.04	0.31
Nodule density	Complete solid nodules	35	33 (94.29)	26 (74.29)	5.29	<0.05
	Part solid nodules	55	53 (96.36)	50 (90.91)	1.37	0.24
	Ground glass nodules	80	78 (97.50)	52 (65.00)	27.73	<0.05

It was revealed in Table-IV that, the sensitivity, specificity, positive prediction rate, negative prediction rate and accuracy of AI detection were significantly higher than those of radiologists (P<0.05).

**Table-IV:** Diagnostic efficacy of the two methods in identifying lung adenocarcinoma.

Methods	n	Sensitivity	Specificity	Positive prediction rate	Negative prediction rate	Accuracy
AI	170	93.38	94.26	95.24	96.52	96.15
Radiologists	170	78.54	79.31	82.03	75.27	78.38
*χ* ^2^		9.07	9.63	8.30	17.79	14.32
*P*		<0.05	<0.05	<0.05	<0.05	<0.05

## DISCUSSION

Dual source CT examination uses the information of single or multiple images to significantly improve the image resolution, improve the image display quality, and better display the morphological characteristics of mass nodules for small nodular lesions.^19^ While improving the diagnostic accuracy, it still has the characteristics of simple operation and convenience.^20^ AI has developed rapidly in the field of early lung cancer CT screening and diagnosis.^21^ AI-aided diagnosis system can accurately find lung nodules on CT, which not only improves the efficiency of lung nodule detection, but also reduces the clinical reading time of radiologists.^22^ However, there are few studies on the diagnostic value of AI-assisted diagnosis combined with CT in early lung cancer.

As reported previously, MIA is more often a larger, lobulated or irregular, mixed ground-glass nodule with a solid component larger than 5 mm.^23^ Consistently, this study indicated the proportions of patients with clear and regular lesion boundaries in AIS were significantly higher than those in patients with MIA, and the cases of mixed ground glass nodules in MIA patients were more than those in AIS patients. Besides, our results suggested that compared with MIA patients, the mean lesion diameter and the CT value of the solid nodule density area of AIS patients were significantly lower. Likewise, a study proposed by Julien G Cohen et al. has indicated that MIA has a solid component size.^24^ In addition, the incidence of pulmonary vascular abnormality, air bronchogram sign as well as pleural depression in lung AIS patients were lower compared to MIA patients. In line with our results, it has been reported that compared with the lung AIS, MIA often has an abnormality in pulmonary vascular, air bronchus and pleural depression.^25^ All these results indicated that AI-based dual source CT scanning may be a good way to differentiate lung AIS from MIA. The reason may be that most of the ultra-high resolution CT images are round pure ground glass nodules with clear boundaries, that is, the focal ground glass shadows on the CT lung window, with uneven density, and air bronchogram sign and pleural depression sign can be seen in a few cases.^26^ In addition, AI-based dual source CT imaging mostly shows mixed ground glass nodules, which are generally membranous glass nodules less than 30 mm and solid components less than 5 mm. These are obviously different from the AI-based dual source CT imaging of lung adenocarcinoma in situ, which can differentiate the two.^27^

In addition, our study compared the sensitivity, specificity and accuracy of the AI system and radiologists’ analysis for the diagnosis of lung adenocarcinoma. Both AI and imaging physicians had high sensitivity for detecting nodules with diameter >5 mm and part solid nodules, which indicated that the detection rate of large nodules, especially nodules with malignant signs, was similar between AI and radiologists. For nodules smaller than 5 mm, complete solid nodules and ground glass nodules, the sensitivity of AI detection was significantly higher than that of radiologists. This may be because that AI automatically extracts image features through deep learning, obtains its three-dimensional information, and identifies and classifies nodules, and currently AI pays more attention to detecting positive nodules. The results of this study showed that specificity, positive prediction rate, negative prediction rate and accuracy of AI detection were significantly higher than those of radiologists, indicating that AI had an ideal detection rate of lung adenocarcinoma, which was consistent with previous literatures.^22^,^28^

### Limitations

It includes the relatively limited sample size. Moreover, a logistic regression analysis was not performed to assess the association of AI quantification-related parameters and lung cancer. The third limitation is that we cannot exclude the mistake on diagnosis from radiologists, despite that they are professional.

## CONCLUSION

Our study indicates compared with MIA patients, AIS patients have clear and regular lesion boundaries, lower mean lesion diameter and the CT value of the solid nodule density area, as well as reduced incidence of pulmonary vascular abnormality, air bronchogram sign and pleural depression through AI-based dual source CT scanning. Our study may be helpful for the differential diagnosis of AIS and MIA in the lung, so that doctors can understand the patient’s condition and develop a more targeted follow-up treatment plan.

### Author’s Contribution:

**LL and JS:** Concept of the study.

**ZN and JZ:** data collection and management.

**JZ:** Data analysis of the study.

**LL and ZN:** First draft.

**JS:** Final revision and responsible and accountable for the accuracy or integrity of the work.

## References

[1] Bade BC, Dela Cruz CS (2020). Lung Cancer 2020:Epidemiology, Etiology, and Prevention. Clin Chest Med.

[2] Nasim F, Sabath BF, Eapen GA (2019). Lung Cancer. Med Clin North Am.

[3] Herbst RS, Morgensztern D, Boshoff C (2018). The biology and management of non-small cell lung cancer. Nature.

[4] Chen Z, Teng X, Zhang J, Huang K, Shen Q, Cao H (2019). Molecular features of lung adenocarcinoma in young patients. BMC Cancer.

[5] Perez-Johnston R, Araujo-Filho JA, Connolly JG, Caso R, Whiting K, Tan KS (2022). CT-based radiogenomic analysis of clinical Stage I lung adenocarcinoma with histopathologic features and oncologic outcomes. Radiology.

[6] Park S, Park G, Lee SM, Kim W, Park H, Jung K (2021). Deep learning-based differentiation of invasive adenocarcinomas from preinvasive or minimally invasive lesions among pulmonary subsolid nodules. Eur Radiol.

[7] Nie M, Yao K, Zhu X, Chen N, Xiao N, Wang Y (2021). Evolutionary metabolic landscape from preneoplasia to invasive lung adenocarcinoma. Nat Commun.

[8] Jiang H, Li X (2017). Correlation of dual-source computed tomography/dual-energy imaging with pathological grading of lung adenocarcinoma and its clinical value. Pak J Med Sci.

[9] Wu G, Woodruff HC, Shen J, Refaee T, Sanduleanu S, Ibrahim A (2020). Diagnosis of Invasive Lung Adenocarcinoma Based on Chest CT Radiomic Features of Part-Solid Pulmonary Nodules:A Multicenter Study. Radiology.

[10] Kuriyama K, Yanagawa M (2020). CT diagnosis of lung adenocarcinoma:radiologic-pathologic correlation and growth rate. Radiology.

[11] Schmidt B, Flohr T (2020). Principles and applications of dual source CT. Phys Med.

[12] May MS, Wiesmueller M, Heiss R, Brand M, Bruegel J, Uder M (2019). Comparison of dual- and single-source dual-energy CT in head and neck imaging. Eur Radiol.

[13] Li T, Zhao S, Liu J, Yang L, Huang Z, Li J (2017). Feasibility of high-pitch spiral dual-source CT angiography in children with complex congenital heart disease compared to retrospective-gated spiral acquisition. Clin Radiol.

[14] Zhang K, Chen K (2022). Artificial intelligence:opportunities in lung cancer. Curr Opin Oncol.

[15] Krarup MMK, Krokos G, Subesinghe M, Nair A, Fischer BM (2021). Artificial Intelligence for the Characterization of Pulmonary Nodules, Lung Tumors and Mediastinal Nodes on PET/CT. Semin Nucl Med.

[16] Jungblut L, Blüthgen C, Polacin M, Messerli M, Schmidt B, Euler A (2022). First Performance Evaluation of an Artificial Intelligence-Based Computer-Aided Detection System for Pulmonary Nodule Evaluation in Dual-Source Photon-Counting Detector CT at Different Low-Dose Levels. Invest Radiol.

[17] Yotsukura M, Asamura H, Motoi N, Kashima J, Yoshida Y, Nakagawa K (2021). Long-term prognosis of patients with resected adenocarcinoma in situ and minimally invasive adenocarcinoma of the lung. J Thorac Oncol.

[18] Obuchowski NA, Bullen JA (2018). Receiver operating characteristic (ROC) curves:review of methods with applications in diagnostic medicine. Phys Med Biol.

[19] Shan Y, Yin X, Zhao N, Wang J, Yang S (2021). Comparison of serum tumor markers combined with dual-source CT in the diagnosis of lung cancer. Minerva Med.

[20] Goo HW (2010). Initial experience of dual-energy lung perfusion CT using a dual-source CT system in children. Pediatr Radiol.

[21] Tunali I, Gillies RJ, Schabath MB (2021). Application of radiomics and artificial intelligence for lung cancer precision medicine. Cold Spring Harb Perspect Med.

[22] Liang F, Li C, Fu X (2021). Evaluation of the Effectiveness of Artificial Intelligence Chest CT Lung Nodule Detection Based on Deep Learning. J Healthc Eng.

[23] Gao F, Ge XJ, Li M, Chen Y, Lyu F, Hua Y (2014). CT diagnosis of different pathological types of ground-glass nodules. Zhonghua Zhong Liu Za Zhi.

[24] Cohen JG, Reymond E, Lederlin M, Medici M, Lantuejoul S, Laurent F (2015). Differentiating pre- and minimally invasive from invasive adenocarcinoma using CT-features in persistent pulmonary part-solid nodules in Caucasian patients. Eur J Radiol.

[25] Zhang Y, Qiang JW, Ye JD, Ye XD, Zhang J (2014). High resolution CT in differentiating minimally invasive component in early lung adenocarcinoma. Lung Cancer.

[26] Sun J, Liu K, Tong H, Liu H, Li X, Luo Y (2021). CT Texture Analysis for Differentiating Bronchiolar Adenoma, Adenocarcinoma In Situ, and Minimally Invasive Adenocarcinoma of the Lung. Front Oncol.

[27] Yan H, Hua Y, Zhang T, Liu W (2022). Differential Diagnosis of Preinvasive Lesions in Small Pulmonary Nodules by Dual Source Computed Tomography Imaging. Comput Math Methods Med.

[28] Zhang Y, Jiang B, Zhang L, Greuter MJW, de Bock GH, Zhang H (2022). Lung Nodule Detectability of Artificial Intelligence-assisted CT Image Reading in Lung Cancer Screening. Curr Med Imaging.

